# *De novo* transcriptome of the mayfly *Cloeon viridulum* and transcriptional signatures of Prometabola

**DOI:** 10.1371/journal.pone.0179083

**Published:** 2017-06-21

**Authors:** Qin Si, Juan-Yan Luo, Ze Hu, Wei Zhang, Chang-Fa Zhou

**Affiliations:** The Key Laboratory of Jiangsu Biodiversity and Biotechnology, College of Life Sciences, Nanjing Normal University, Nanjing, China; Xiamen University, CHINA

## Abstract

Mayflies (Ephemeroptera) display many primitive characters and a unique type of metamorphosis (Prometabola). However, information on the genomes and transcriptomes of this insect group is limited. The RNA sequencing study presented here generated the first *de novo* transcriptome assembly of *Cloeon viridulum* (Ephemeroptera: Baetidae), and compared gene expression signatures among the young larva (YL), mature larva (ML), subimago (SI), and imago (IM) stages of this mayfly. The transcriptome, based on 88 Gb of sequence data, comprised a set of 81,185 high quality transcripts. The number of differentially expressed genes (DEGs) in YL vs. ML, ML vs. SI, and SI vs. IM, was 4,825, 1,584, and 1,278, respectively, according to the reads per kilobase of transcript per million mapped reads analysis, assuming a false discovery rate <0.05 and a fold change >2. Gene enrichment analysis revealed that these DEGs were enriched in the “chitin metabolic process”, “germ cell development”, “steroid hormone biosynthesis”, and “cutin, suberine, and wax biosynthesis” pathways. Finally, the expression pattern of a selected group of candidate signature genes for Prometabola, including vestigial, methoprene-tolerant, wingless, and broad-complex were confirmed by quantitative real time-PCR analysis. The Q-PCR analysis of larval, subimaginal, and imaginal stages of *C*. *viridulum* suggests that the development of mayflies more closely resembles hemimetamorphosis than holometamorphosis.

## Introduction

Mayfly (Ephemeroptera) fossil records have more than 300 million years. The living species show many primitive and unique characteristics of winged insects, including a four-stage (Prometabola) life history (egg, larva, subimago, and imago), unfolded wings, obvious corrugation on wings, long segmented tails, and nymphs with paired gills. Among these characteristics, the prometabolan pattern with two winged instars (subimago and imago) is the most primitive metamorphosis found in Pterygota and it exists exclusively in Ephemeroptera. The origin of Prometabola and functions of subimagos have been extensively discussed [1]. However, the key questions concerning the role of the subimago and subimagos change in Prometabola remain unsolved. Chen [2] and Tan [3] postulated that the subimaginal stage of mayflies is equivalent to the pupal stage of Holometabola (i.e., insects with complete metamorphosis); when lost during evolution, the incomplete metamorphosis pattern was originated. Schaefer [4] and Kukalová-Peck [5], however, suggested that the subimaginal stage is a relic of one or more imaginal instars of primitive insects, presumably resulting from the preservation or compression of one or more molt processes into one stage during evolution. However, all these postulations were based on morphological and ecological comparisons, and molecular studies remain to be performed.

Using microarray methods, Holometabola development has been studied in several species, such as the fruit fly [6], ant [7], and silkworm [8]. Next-generation sequencing (NGS) based on the RNA sequencing (RNA-seq) technique has been widely used as a comprehensive approach to study the genome and transcriptome of a broad range of organisms, generating information on many insect transcriptomes including locust (*Locusta migratoria*) [9], field cricket (*Gryllus bimaculatus*) [10], dragonflies (*Enallagma hageni* and *Ischnura elegans*) [11, 12], mosquito (*Anopheles gambiae*) [13], flour beetle (*Tribolium castaneum*) [14], common cutworm (*Spodoptera litura*) [15], Chinese white wax scale (*Ericerus pela*) [16], and fruit fly (*Drosophila melanogaster*) [17, 18]. However, no transcriptome assembly of mayflies has been published.

**Fig 1:**
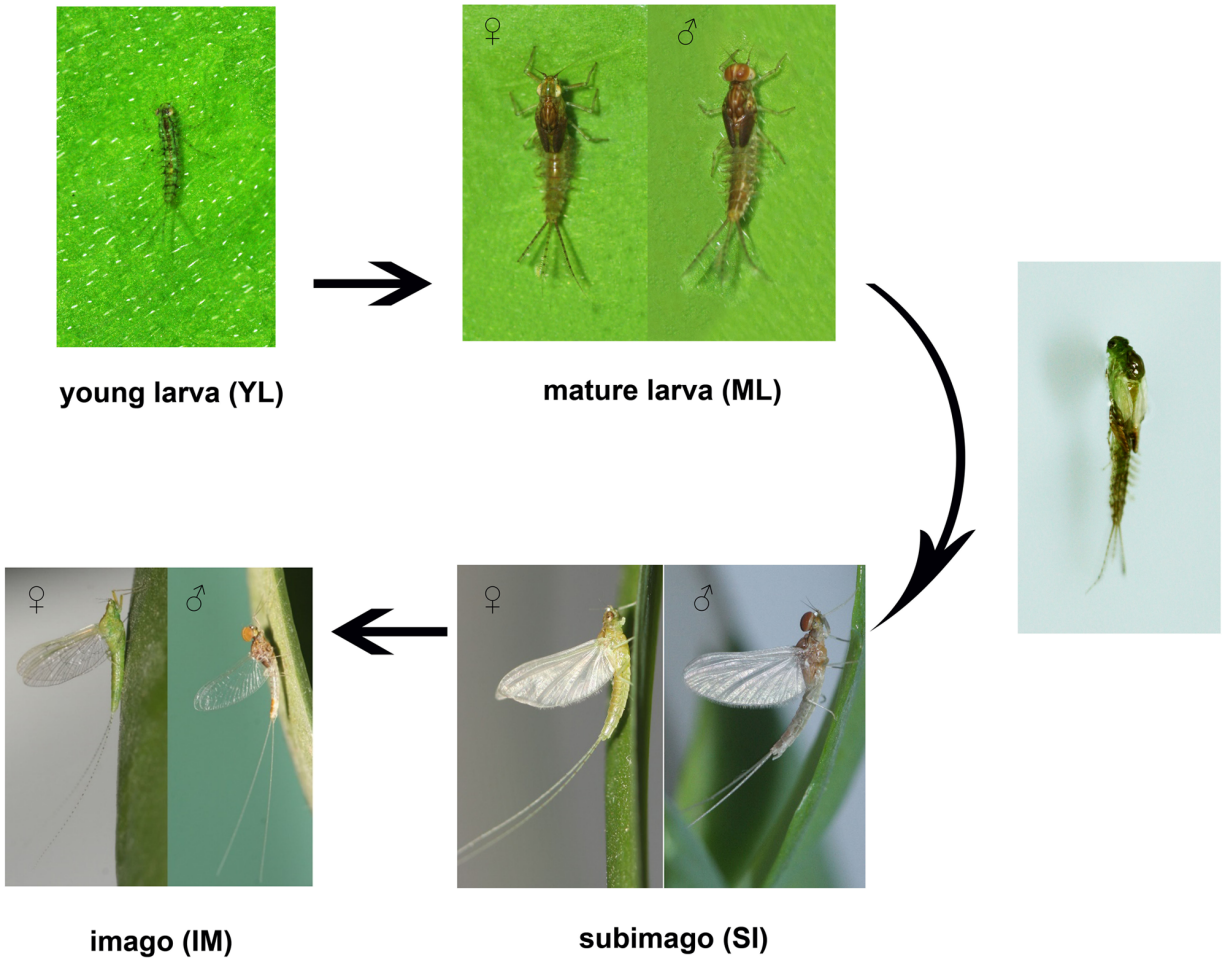
*Cloeon viridulum* at different developmental stages. The young larva (nymph, without wingpad; YL), mature larva (with wingpad; ML), subimago (SI), and imago (IM) stages of *C*. *viridulum*, which endure two molting process from ML to IM.

In the present study, RNA-seq [19, 20] was applied to comprehensively characterize the global gene expression pattern of the mayfly *Cloeon viridulum* in different life stages, aiming to provide a genetic basis for understanding the metamorphosis, origin, and phylogeny of winged insects. Fifteen cDNA libraries, based on three biological replicates, were established for the four critical stages of this species: young larva (without wingpads; YL), mature larva (with dark wingpads; ML), subimago (SI), imago (IM), and mixture sampling (composite group, CG) (Fig 1). Furthermore, candidate signature genes that are involved in the development of Prometabola were identified and analyzed.

## Results

### Sequencing and *de novo* assembly of *Cloeon viridulum*

To identify mRNA differentiation in *C*. *viridulum*, 15 cDNA libraries were established, including three young larva groups (marked as YL1, YL2, and YL3), three mature larva groups (ML1, ML2, and ML3), three subimaginal groups (SI1, SI2, and SI3), three imaginal groups (IM1, IM2, and IM3), and three composite groups (made up from YL, ML, SI and IM: CG1 CG2, and CG3). The 750,960,878 raw reads generated had Q20 (the sequencing quality value corresponding to 1% chance of error) values above 97% (S1 Table). After removing adapters and trimming low-quality and ambiguous reads, 733,069,734 high-quality and clean reads were obtained (S2 Table).

In the *de novo* assembly carried out using CLC Genomics Workbench v6.0.4 (CLC Bio, Aarhus, Denmark), reads from the 15 samples were pooled to assemble 140,513 contigs with an N50 (contig length for which half the assembly contains contigs of this size or longer) of 812 bp, average length of 629 nucleotides, and the longest sequence of 15,030 bp. These contigs were further assembled into 94,580 scaffolds (average length = 850 bp) with N50 = 1,179 bp. After clustering to other animals, using the software CAP3 EST [21], 81,185 unigenes (scaffolds that cannot be extended on either end) with a mean length of 905 bp (contig N50 = 2,992 bp) (S3 Table) were generated, comprising 73,440,746 bp in total. The number of unigenes whose length is above 1,000 bp was 17,742 (S1 Fig). The high average and median unigene lengths suggested that many full-length transcripts were successfully assembled.

### Functional annotation

The 81,185 assembled unigenes were queried against the UniProt (including SWISS-PROT and TrEMBL), non-redundant NCBI (Nr), Gene Ontology (GO), EuKaryotic Orthologous Groups (KOG), and Kyoto Encyclopedia of Genes and Genomes (KEGG) databases using the basic local alignment search tool (BLAST), resulting in 30,027 (36.99%), 37,878 (46.66%), 11,056 (13.62%), 15,763 (19.42%), and 7,199 (8.87%) gene matches, respectively (S4 Table). The BLAST result shown in S2 Fig evidence that the species with most hits (17.90%) was incomplete metabolan insect *Zootermopsis nevadensis* (Isoptera).

Gene Ontology analysis showed that 11,056 non-redundant genes corresponded to at least one GO term, which could be categorized into 52 functional groups (S3 Fig). Genes within the three main GO categories, i.e., biological process (16,316 unigenes), cellular component (8,003 unigenes), and molecular function (25,970 unigenes), were mainly assigned to the subcategories ‘‘behavior”, ‘‘molecular transducer activity”, and ‘‘single−organism process”, respectively. The ‘‘extracellular region”, ‘‘immune system process”, ‘‘metabolic process”, “membrane−enclosed lumen”, and ‘‘synapse part” subcategories were also well represented, whereas few genes were assigned to ‘‘detoxification”, “growth”, or “supramolecular fiber”.

To further evaluate transcriptome completeness and the effectiveness of the annotation process, a KOG classification was conducted resulting in the assignment of 15,763 unigenes to at least one KOG category (S4 Fig). Among the 25 KOG categories, the cluster for “general function prediction only” (2,142, 13.59%), followed by “signal transduction mechanisms” represented the largest group (2,132 unigenes, 13.53%), “posttranslational modification, protein turnover, chaperones” (2,124, 13.47%), and “Transcription” (2,051, 13.01%). The “Nuclear structure” (279, 1.77%), “defense mechanisms” (278, 1.76%), “coenzyme transport and metabolism” (238, 1.51%), and “cell motility” (48, 0.30%) categories were the least annotated. These results provide a valuable molecular basis to further investigate specific processes and functions in *C*. *viridulum*.

Unigenes were further mapped to the biological pathways involved in the development of *C*. *viridulum*, recorded in the KEGG database. The KEGG classifications for 7,199 genes are five main biochemical pathways (S5 Fig). Dominant pathway categories for them, “organismal systems” (1,881), “metabolism” (2,550), “genetic information processing” (2,232), “environmental information processing” (1,532), and “cellular processes” (1,529) were mainly annotated in the “endocrine system” (770, 40.94%), “carbohydrate metabolism” (750, 29.41%), “translation” (1,041, 46.64%), “signal transduction” (1,352, 88.25%), and “transport and catabolism” (818, 53.50%) pathways, respectively. Overall, the 7,199 unigenes were categorized into 336 known KEGG pathways, being “metabolic” (2,100 unigenes), “Biosynthesis of secondary metabolites” (770), and “Biosynthesis of antibiotics” (534) the top three pathways containing the most unigenes.

### Transcriptional changes among developmental stages

**Fig 2:**
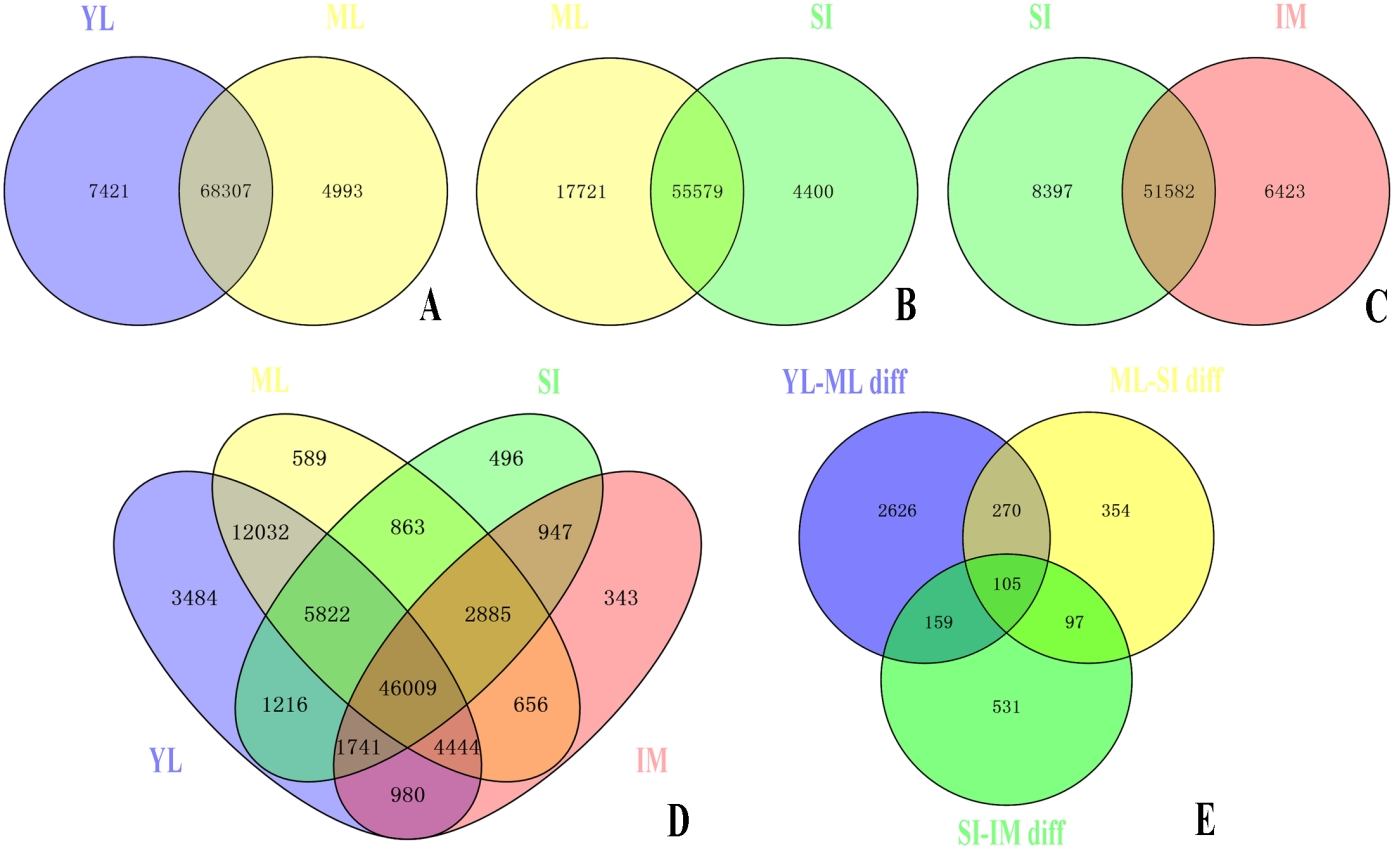
Changes in the transcriptome of *Cloeon viridulum* through its developmental stages. Genes associated with a q-value of less than or equal to 0.05 for at least one stage were used to construct Venn diagrams of the differentially expressed genes (DEGs) in (A) young larva (YL) and mature larva (ML), (B) in ML and subimago (SI), (C) in SI and imago (IM), (D) at the four developmental stages, and (E) in YL vs. ML, ML vs. SI, and SI vs. IM.

**Fig 3:**
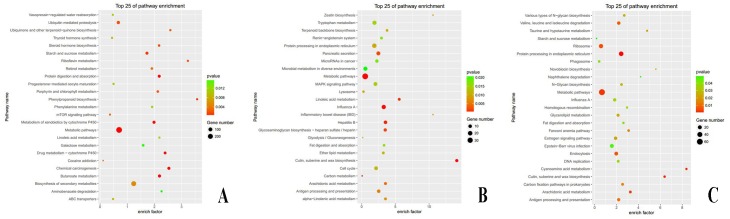
Kyoto Encyclopedia of Genes and Genomes (KEGG) pathway enrichment analyses of DEGs obtained from RNA sequencing (25 pathways). KEGG pathway enrichment analyses of DEGs (A) between young larva (YL) and mature larva (ML), (B) between ML and subimago (SI), and (C) between SI and imago (IM) stages.

Differences in gene expression among the four stages of mayfly development were examined and differentially expressed genes (DEGs) were identified by pairwise comparisons seriatim (YL vs. ML, ML vs. SI, and SI vs. IM) and globally (YL vs. ML vs. SI vs. IM) (Fig 2). The YL group had the largest number of specific DEGs (3,484). There were 4,825 DEGs between the YL and ML libraries, with 1,011 up- and 3,814 down-regulated genes in ML (S6 Fig). In GO and KEGG categories, these DEGs were related to important biological processes such as growth and development, including the GO terms “micropinocytosis”, “translation”, “cuticle pigmentation”, and “melanin biosynthetic process”, and the KEGG pathways “steroid hormone biosynthesis”, “metabolism of xenobiotics by cytochrome P450”, and “protein digestion and absorption” (Fig 3).

The number of DEGs decreased to 1,584 (1,265 up-regulated and 319 down-regulated genes) when ML was compared to SI (S6 Fig). Most biological process terms were related to “chitin” and gene expressions were largely up-regulated in SI, which is probably related to the cuticle development during ecdysis to and within the SI stage. In addition, DEGs were enriched in some important GO terms, namely “chitin metabolic process”, “chitin-based cuticle development”, “carbohydrate metabolic process”, and “macropinocytosis”, and KEGG pathways, namely “cutin, suberine, and wax biosynthesis”, “pancreatic secretion”, and “fat digestion and absorption” (Fig 3), which were closely linked to metamorphosis.

The number of DEGs between SI and IM slightly declined to 1,278 (S6 Fig), with 377 down-regulated genes (mainly linked to the GO terms “chitin metabolic process” and “chitin-based cuticle development”) and 901 up-regulated genes (enriched in the “germ cell development”, “spermatocyte division”, “embryo development”, “oogenesis”, “peripheral nervous system development”, “learning or memory”, and “olfactory learning” terms). The KEGG pathways “protein processing in endoplasmic reticulum”, “cutin, suberine, and wax biosynthesis”, “fat digestion and absorption”, and “estrogen signaling pathway” were found to be linked to these DEGs (Fig 3).

**Fig 4:**
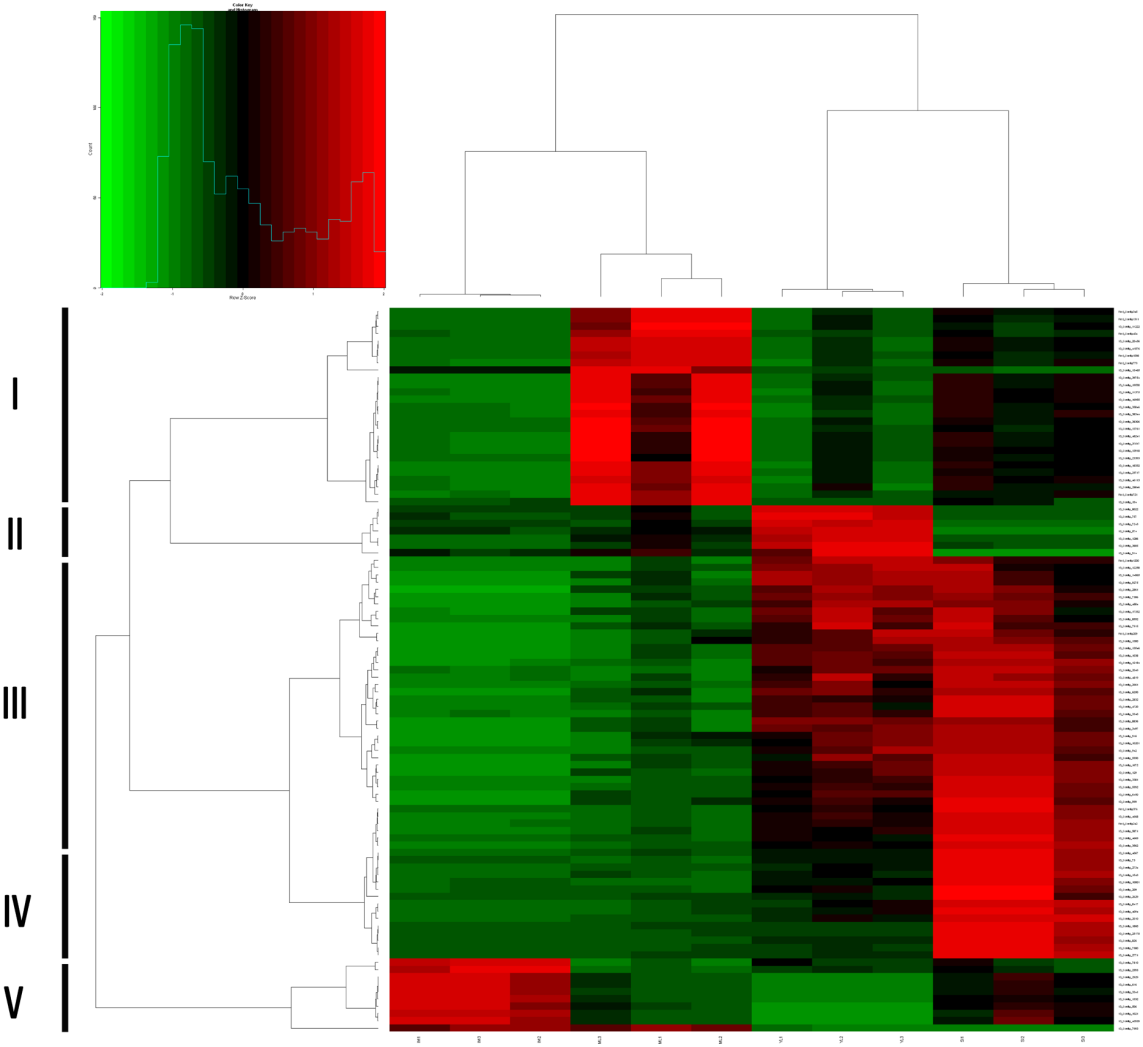
Hierarchical clustering of 100 differentially expressed gens (DEGs) at the four developmental stages of *Cloeon viridulum*. Clustering was based on the log_2_ (reads per kilo of per million mapped reads + 1) obtained from the RNA sequencing data of 12 samples (young larva (YL)1, YL2, YL3, mature larva (ML)1, ML2, ML3, subimago (SI)1, SI2, SI3, and imago (IM)1, IM2, IM3). Based on their expression pattern, genes are divided into five clusters: clusters I, II, IV, and V contain genes expressed on ML, YL, SI, and IM respectively, while the genes in cluster III are expressed in both YL and SI.

The hierarchical clustering of 100 DEGs, based on the log_2_ [reads per kilobase of transcript per million mapped reads (RPKM) + 1] of 12 samples showed that these genes could be divided into five clusters, most with uniquely expressed genes; genes in cluster III were expressed in both YL and SI samples. These results revealed that differences in gene expression during *C*. *viridulum* development were remarkable and that the selected samples could be sorted into four distinct groups based on different stages (Fig 4), which was also evidenced in the Venn diagram produced for the distribution of different genes between each pair of stages (p-value cut off of 0.05, Fig 2D). These results indicated that the selected stages were the most valuable instars for the study of mayflies’ metamorphosis, and that the genes involved in the annotated biological processes may play crucial roles in the metamorphosis of *C*. *viridulum*.

### Quantitative real time-PCR validation of significant DEGs

**Fig 5:**
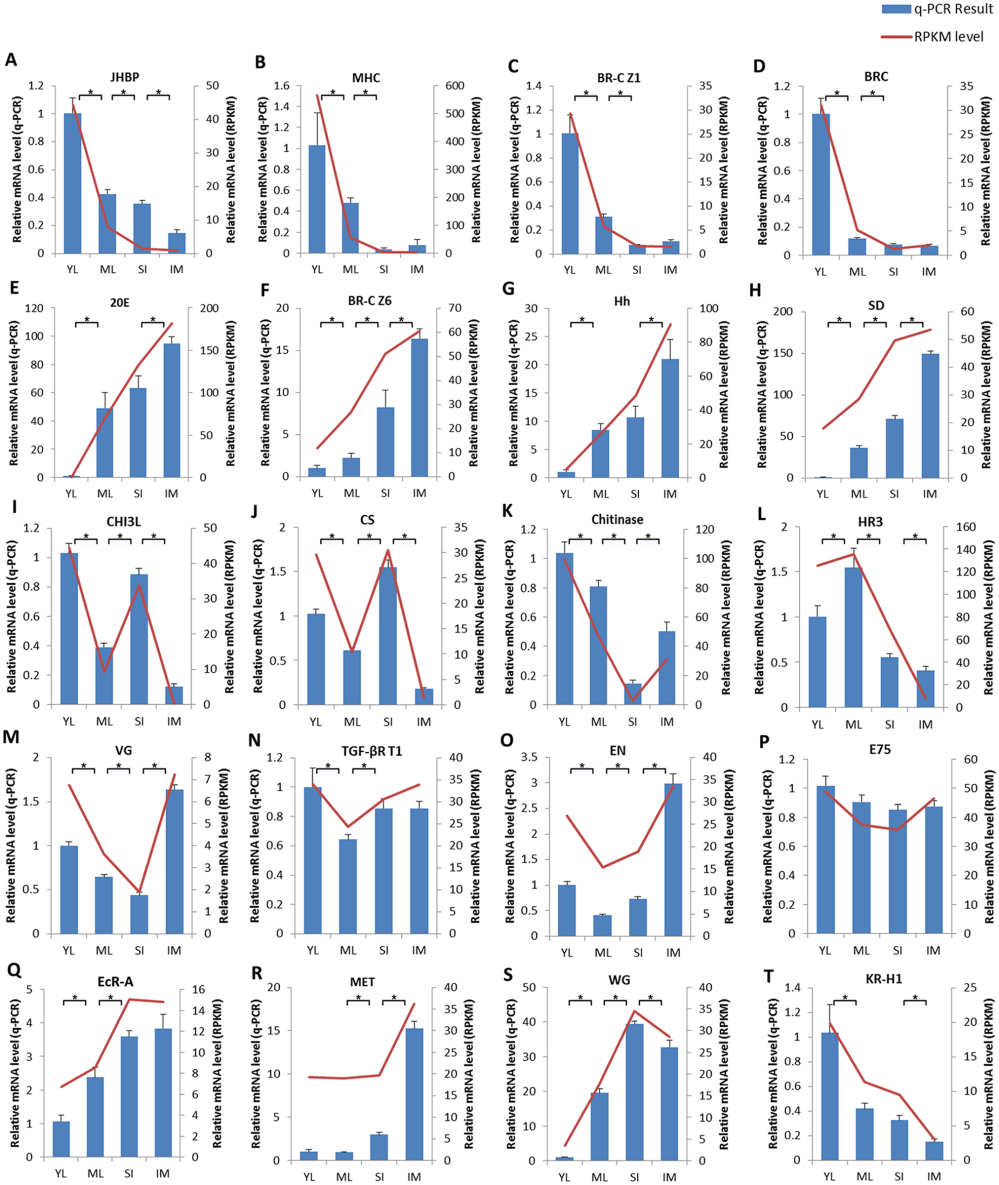
Expression of the selected DEGs putatively involved in metamorphosis. The relative abundance of 20 selected genes at the four stages of *Cloeon viridulum* development, namely young larva (YL), mature larva (ML), subimago (SI), and imago (IM), was examined using Illumina RNA-sequencing (red line) and quantitative real-time PCR (blue columns). **A**: Hemolymph juvenile hormone binding protein (JHBP); **B**: Myosin heavy chain (MHC); **C**: Broad-complex isoform Z1 (BR-C Z1); **D**: Broad-complex (BR-C); **E**: Ecdysone 20-monooxygenase (20E); **F**: Broad-complex core protein isoform 6 (BR-C Z6); **G**: Hedgehog (Hh); **H**: Scalloped (Sd); **I**: Chitinase-3-like (CHI3L); **J**: Chitin synthase (CS); **K**: Chitinase (CA); **L**: Nuclear hormone receptor HR3 (HR3); **M**: Vestigial (Vg); **N**: TGF-beta receptor type-1 (TGF-βR T1); **O**: Engrailed (En); **P**: Ecdysone-inducible protein E75 (E75); **Q**: Ecdysone receptor A isoform (EcR-A); **R**: Methoprene-tolerant (Met); **S**: Wingless (Wg); **T**: Kruppel-like protein 1 (Kr-h1). Relative expression levels were normalized to *CvGAPDH* levels. Values are means ±standard error of the mean (n = 3). Asterisks (*) indicate statistical significance (p < 0.05).

The expression profiles of 20 DEGs were further validated using quantitative real time (qRT)-PCR. These DEGs, which were manually selected as representatives due to their potential roles in Prometabola according to the annotations performed, encoded: hemolymph juvenile hormone binding protein (JHBP), myosin heavy chain (MHC), broad-complex isoform Z1 (BR-C Z1), broad-complex (BR-C), ecdysone 20-monooxygenase (20E), broad-complex core protein isoform 6 (BR-C Z6), hedgehog (Hh), scalloped (Sd), chitinase-3-like (CHI3L), chitin synthase (CS), chitinase (CA), nuclear hormone receptor HR3 (HR3), vestigial (Vg), TGF-beta receptor type-1 (TGF-βR T1), engrailed (En), ecdysone-inducible protein E75 (E75), ecdysone receptor A isoform (EcR-A), methoprene-tolerant (Met), wingless (Wg), and Kruppel-like protein 1 (Kr-h1). Most mRNAs showed a similar expression tendency to that obtained in RNA-Seq (RPKM/reads-based expression values) (Fig 5 and S5 Table), suggesting that the RNA-Seq data are reproducible and reliable.

## Discussion

### GO and KEGG enrichment analysis of DEGs in YL vs. ML

The four stages of mayflies differ morphologically in their wings, eyes, gills, and other structures. During the aquatic larval stages (i.e., from YL to ML), mayflies change their body size, gills, eyes, terga, color, and wingpads significantly. Notably, most DEGs down-regulated in ML compared to YL were linked to the GO terms “micropinocytosis”, “translation”, and “cuticle pigmentation”. Micropinocytosis and translation provide a material base for insects’ growth. Macropinocytosis, which serves additional physiological functions, is a form of endocytosis that accompanies cell surface ruffling. Virtually all cells use endocytosis to absorb nutrients from the surrounding environment and to regulate the expression of cell surface molecules [22]. Translation is the cellular metabolic process in which a protein is formed. Cuticle pigmentation is responsible for the establishment of pigmentation patterns in the cuticle of an organism, forming the special color observed in mayflies. Pigmentation/melanization (tanning) is an important physiological event in cuticle formation, wound healing, and encapsulation during the defensive response to parasite invasion as well as in the hardening of the egg chorion [23]. Additionally, DEGs were notably enriched in the “metabolism of xenobiotics by cytochrome P450” and “steroid hormone biosynthesis” pathways. These two pathways are related to the regulation of Cytochrome P450 enzymes, which is present in all living organisms and involved in the metabolism of endogenous substrates (steroid hormones, lipids, etc.), and in the regulation of steroid hormones, which participate in organ development, reproduction, body homeostasis, and stress responses [24]. The P450 enzymes fulfill many important tasks, from the synthesis and degradation of ecdysteroids and juvenile hormones to the metabolism of foreign chemicals of natural or synthetic origin [25]. In these pathways, Glutathione S-transferase, Juvenile hormone epoxide hydrolase 1, UDP-glucuronosyltransferase 2C1, Cytochrome P450 2J3, Cytochrome P450 9E1, Cytochrome P450 6BQ11, Cytochrome P450 CYP6BW5v1, Steroid dehydrogenase, Cytochrome P450 CYP15A1, Estradiol 17-beta-dehydrogenase 12, Cytochrome P450 6BQ8, and Cytochrome P450 2J3 expression were significantly down-regulated, also evidencing the down-regulation of steroid hormone biosynthesis and metabolism of xenobiotics by the Cytochrome P450 signaling pathway.

### GO and KEGG enrichment analysis between ML and SI stages

When mayflies molt from ML to SI, they stretch their membranous wings, develop their compound eyes and genitalia, loose their gills, slightly degrade their mouthparts, and, most importantly, leave water to live on land or in air. Most of the DEGs enriched in the GO terms “chitin metabolic process” and “chitin-based cuticle development”, which are closely related to the molting process, were remarkably up-regulated in SI compared to ML. This enrichment suggested that chitin synthesis and degradation was rapidly metabolized during the SI stage and provided molecular materials for molting. Previous studies have illustrated that the insect cuticle is mainly composed of chitin-combining proteins that contain chitin-binding domains [26]. Further, the cuticular protein, which is analogous to peritrophin and serves essential and non-redundant functions in maintaining the structural integrity of the cuticle in different parts of the insect body [27], was significantly up-regulated in the present study. The “cutin, suberine, and wax biosynthesis” and “pancreatic secretion” pathways also changed dramatically from ML to SI, indicating that cuticle formation and digestive ability is increased during *C*. *viridulum* development. The pancreas performs both exocrine and endocrine functions. The exocrine pancreas consists of acinar and duct cells, and the primary functions of pancreatic acinar cells are to synthesize and secrete digestive enzymes. This might be related to energy generation and maintenance in adult mayflies as they have lost their mouthparts in the last two life stages. The transcriptional expressions of several enzymes (e.g., phospholipase A2D, phospholipase A1 member A, serine protease, Carboxypeptidase, and cAMP-dependent protein kinase) were also significantly changed in some pathways, indicating that they might play important roles in the SI stage.

Some genes, such as bone morphogenetic protein genes (*BMPs*) [28, 29], *TGF-beta* [30], *Vg* [31], *En* [32], and *Hh* [33] genes, are necessary to the growth and patterning of *Drosophila* sp. wings. These genes were also significantly up-regulated in the transition from ML to SI in *C*. *viridulum*, which is not surprising given that wing development is a major achievement during the SI stage of mayflies. Similarly, some *Wnt* genes (like Wingless/WNT1) were considerably up-regulated in SI. Previous researches have drawn the conclusion that these genes are closely related to the wing development and adult metamorphosis of *Tribolium* [34] and *Drosophila* [35].

### GO and KEGG enrichment analysis between SI and IM stages

The character that differentiates mayflies from other insects is their need to endure molting from subimago to imago to develop their reproductive system and ability, sensory organs, and copular structures. The DEGs up-regulated from SI to IM were mainly linked to GO terms concerning reproduction and nervous system development, such as “germ cell development”, “spermatocyte division”, “embryo development”, “oogenesis”, “peripheral nervous system development”, “learning or memory”, and “olfactory learning”, whereas the down-regulated DEGs were mainly linked to the GO terms “chitin metabolic process” and “chitin-based cuticle development”, which was quite different from that observed from ML to SI. Compared to SI, the IM DEGs were only remarkably enriched in the “protein processing in endoplasmic reticulum” KEGG pathway. This pathway packages correctly folded proteins to the Golgi complex and retains misfolded proteins with molecular chaperones or degrades the terminally misfolded proteins through the proteasome, acting upon heat-shock protein family members, protein disulfide-isomerase [36], and dual specificity mitogen-activated protein kinase kinase 7 [37]. These findings indicated that IM is slightly different from SI, mainly in its developed nerves, reproduction ability and inhibited their aging especially. The molecular analysis performed here confirms the morphological and biological transformations from SI to IM.

### Crucial genes involved in Prometabola

The two main developmental hormones in insects are ecdysones (belonging to the hydroxylated steroid hormones family) and juvenile hormones (JHs, which belong to the sesquiterpene hormones family) [38]. In general, ecdysones are molting hormones, with periodic surges causing the events involved in molting, whereas JHs are status-quo hormones that maintain the insect in its current form as the insect is responding to the molting surge of ecdysone [39]. Two hormones are mainly involved in insect metamorphosis: 20E, which promotes the successive molts that allow growth and postembryonic development, and juvenile hormone (JH), which represses metamorphosis [40]. In hemimetabolous insects, the JH titer drops to undetectable levels at the onset of the last nymphal stage and the next ecdysone surge leads to the adult stage. In Holometabola, in contrast, JH declines at some point in the last larval stage and the following small surge of ecdysone terminates larval feeding, promoting premetamorphic behaviors, such as cocoon spinning, and committing larval tissues to pupal development [40, 41]. The cuticle of the pupal stage is produced in response to a second large surge of ecdysteroids, which is associated with the transient re-appearance of JH. This hormone is eliminated just before pupal ecdysis so that the subsequent surge of ecdysone in the pupal stage causes adult commitment and differentiation. In the present study, the expression profile of 20E increased consistently from young larva to imago, while the Juvenile Hormone epoxide hydrolase 1 and Juvenile Hormone esterase-like protein Est1 decreased constantly. This pattern is similar to hemimetabolous rather than to holometabolous insects.

Some studies found that JH might exhibit its functions through two transcription factors, ecdysone-inducible protein 75 isoform A (E75A) [42, 43] and Kr-h1 [44, 45], to regulate the activation of BR-C [46]. In hemimetabolous species, JH stimulates BR-C transcription during early nymphal stages, which weakened in the last nymphal instar when circulating JH vanished [47, 48]. In contrast, in holometabolous insects, JH inhibits BR-C expression during larval stages, but when forming the pupal stage, JH disappeared and 20E was produced in a small peak. The expression levels of most *br* genes in the mayfly *C*. *viridulum* decreased from early to late instars, which was also similar to the hemimetabolous pattern.

Transcription factors Hormone receptor 3 (HR3) and E75A were reported to be involved in 20E signal transduction and capable of binding with the response element of many cuticle protein genes [49, 50], and may also be involved in regulating the expression of cuticle proteins in the wing disc during metamorphosis [51]. In the present study, E75 changed slightly during the four stages but HR3 only increased slightly in the ML stage of *C*. *viridulum*, in agreement with its role in the regulation of metamorphosis.

Chitin is an important component of cuticle, and its content changes greatly during the metamorphic process. In some studies, chitin content decreased when chitin catabolic enzymes (chitinase and β-N-acetylglucosaminidase) were up-regulated in some holometabolous insects pre-pupae and then increased when chitin synthase was up-regulated in pupae [15]. In the mayfly *C*. *viridulum*, chitin catabolic enzymes (chitinase) were highly expressed in YL and lowly expressed in SI. However, relevant genes for both chitin binding and chitin synthesis were significantly up-regulated in the latter stage, indicating that the SI stage is crucial for chitin synthesis and production in *C*. *viridulum*, similar to the pupa of holometabolous insects.

## Conclusions

The present study provided a *de novo* transcriptome of the mayfly *C*. *viridulum* based on its four developing stages: young larva, mature larva, subimago, and imago. This is the first complete transcriptome assembly of Ephemeroptera and the 88 Gb of sequence data provided 81,185 high-quality transcripts. Comparisons among the molecular data obtained for the four stages identified DEGs that were classified into several crucial GO and KOG categories, as well as in KEGG pathways. Furthermore, some putative genes related to metamorphosis, such as *MHC*, *Vg*, *Met*, *Sd*, *EcR-A*, *Wg*, *BR-C*, and *En*, were identified. Based on the number of DEGs and their changing patterns, the development pattern of mayfly is more similar to hemimetamorphosis than to holometamorphosis. In addition, some key genes or pathways for metamorphosis, like those linked to wing, hormones, and chitin development and synthesis, were only expressed or more up-regulated in the subimaginal stage of mayfly. Thus, transcriptome data, as well as morphological and biological characters, indicate that changing from mature larva to subimago is the most crucial stage in the mayfly life history and the subimaginal stage is an instar of mayfly adults.

## Materials and methods

### Sample collection and preparation

The young *C*. *viridulum* larvae used in this study were collected in the Caiyue pool or lake, Nanjing Normal University, Nanjing, Jiangsu province, eastern China. This species can be found in most of China and is very common. The locality of collection is open to public.

Young larvae were reared in an insectary, at 22 ± 1°C, 60% relative humidity, and under 16:8-h (light:dark) photoperiod, using water and plants from the pool they were collected from. Mature larva, subimagoes, and imagoes were reared from young larvae in the laboratory. Young larvae samples comprised 10 individuals, ML, SI, and IM samples comprised three males and three females each, and CG samples comprised five YL, and two ML, two SI, and two IM, each represented by a male and a female individual. Each sample had three biological replicates (YL1, YL2, YL3; ML1, ML2, ML3; SI1, SI2, SI3; IM1, IM2, IM3; CG1, CG2, CG3). Samples were cryopreserved in liquid nitrogen and stored at -80°C until use.

Total RNA was extracted from each sample using TRIZOL Reagent (Cat#15596–018, Life Technologies, Carlsbad, CA, US), following the manufacturer’s instructions, and their RNA integrity was checked in the Agilent Bioanalyzer 2100 (Agilent Technologies, Santa Clara, CA, US). Qualified total RNA was further purified using the RNeasy micro kit (Cat#74004, QIAGEN, GmBH, Germany) and RNase-Free DNase Set (Cat#79254, QIAGEN).

### Data processing and *de novo* transcriptome assembly

Approximately 1 μg of total RNA per sample was used to construct the corresponding cDNA libraries. The library used in RNA-Seq was generated using the Illumina TruSeq RNA Sample Preparation Kit (Illumina, San Diego, CA, USA). Transcriptome sequencing was carried out by SBC (Shanghai Biotechnology Corporation, Shanghai, China) on the Illumina HiSeq 2500 platform, and approximately 125-bp paired-end raw reads were generated. Reads with low overall quality (proportion of identified bases below 50%), adaptor sequences, ambiguous nucleotides (“Ns”), low quality (quality score below 20), and shorter than 20 nucleotides, and ribosome RNA reads were removed. The remaining clean reads were firstly assembled using CLC Genomics Workbench to obtain the primary unigenes, which were then assembled using CAP3 EST [21] to obtain the final unigenes of real animals, as described for *de novo* transcriptome assemblies without a reference genome. The quality of the assembly was critically assessed by SBC before subsequent analysis.

### Functional annotation and classification

The assembled contigs were annotated by searching against the NCBI Nr and UniProt (SWISS-PROT and TrEMBL) databases using BlastX with E-value < 1e5. The best match results were used to determine the closest known taxonomy of the unigenes, according to their identity percentage, and species scoring the highest blast hit were identified. Based on annotation results, contigs were classified into “biological processes”, “cellular components”, and “molecular functions” GO terms in Blast2GO (http://www.blast2go.de) [52]. In the present study, GO analysis was performed at level 2. The contigs were then queried to the KOG database (http://www.ncbi.nlm.nih.gov/COG/) to classify and predict their functions. In addition, unigenes were subject to KEGG pathways analysis on the KEGG Automatic Annotation Server online. GO and KEGG are the major public databases for further understanding the biological functions of genes as well as the utilities of the biological systems detected in large-scale molecular datasets (http://www.genome.jp/kegg/).

### Analysis of DEGs

The expression level of each unigene was measured as the number of reads mapped to its sequence, which was normalized to RPKM. DEGs were identified following Anders and Huber [53]. The significance threshold in multiple tests was set by the false discovery rate (FDR). A FDR <0.05 and a fold-change ≥2 were used as thresholds to define significantly DEGs.

Gene ontology and KEGG pathways enrichment analysis identifies significantly enriched biological processes and metabolic or signal transduction pathways using the corrected P-value< 0.05 as a threshold to find significantly enriched terms in a list of DEGs, comparing them to the whole genome background. The P-value was calculated as:

$$\text{P}=1-\Sigma_{\text{i}=0}^{\text{m}-1}\frac{\left(\frac{\text{M}}{\text{i}}\right)\left(\frac{\text{N}-\text{M}}{\text{n}-\text{i}}\right)}{\frac{\text{N}}{\text{n}}}$$

where N represented the number of GO/KEGG annotated genes in *C*. *viridulum*, n represented the number of DEGs in N, M represented the number of particular GO/KEGG annotated genes in a genome, and m represented the number of particular GO/KEGG annotated DEGs in M. After Bonferroni correction, we chose the pathways with a P-value < 0.05 to represent those with significantly enriched DEGs.

### qRT-PCR

The expression profiles of 20 DEGs from *C*. *viridulum* were further validated using qRT-PCR. Total RNA was isolated using Trizol reagent (BioTeke, Beijing, China), and cDNA was generated using 1 μg total RNA and a reverse transcription kit (Takara, Dalian, China).

To verify RNA-seq results, a qRT-PCR was performed to explore mRNA expression levels, using *CvGAPDH* as the internal control and the SYBR Green Master Kit (Roche, Basel, Switzerland), according to the manufacturer’s protocol. The primers used in qRT-PCR are listed in S5 Table. Experiments were carried out in triplicate in a total volume of 20 μL containing 10 μL SYBR Green Master mix, 6 μL cDNA (100 ng), and forward and reverse primers (2 μmol/L; 2 μL each). The qRT-PCR was performed on a ABI step one TM plus (Applied Biosystems, Waltham, MA, USA), using the profile: 10 min at 95°C followed by 40 cycles of 15 s at 95°C for plus 1 min at 60°C. The expression level was calculated by the 2^-ΔΔCT^ method, and subjected to statistical analysis [54]. In mRNA expression analysis, RPMK values were determined by comparison with that of the reference gene.

## Supporting information

S1 Fig Length distribution of the assembled unigenes obtained from Illumina sequences of *Cloeon viridulum*. (TIF)

S2 Fig Species distribution. (TIF)

S3 Fig Gene Ontology classification of the assembled unigenes. (TIF)

S4 Fig Clusters of Eukaryotic Orthologous Groups classification. (TIF)

S5 Fig Kyoto Encyclopedia of Genes and Genomes pathways of the differentially expressed genes. (TIF)

S6 Fig Number of differentially expressed genes among developmental stages. (TIF)

S1 Table Summary of the sequence repeats identified in *Cloeon viridulum*. (PDF)

S2 Table Summary of the RNA sequence results obtained for young and mature larvae, subimagos, and imagos. (PDF)

S3 Table Statistical summary of *C*. *viridulum*’s transcriptome assembly. (PDF)

S4 Table Blast analysis of non-redundant unigenes against public databases. (PDF)

S5 Table Primers used in this study. (PDF)
