# Supplementary figures and images for: De novo transcriptome of the mayfly Cloeon viridulum and transcriptional signatures of Prometabola
Source: PLoS One. 2017 Jun 21;12(6):e0179083. doi: 10.1371/journal.pone.0179083 (PMC5479533; doi:10.1371/journal.pone.0179083)

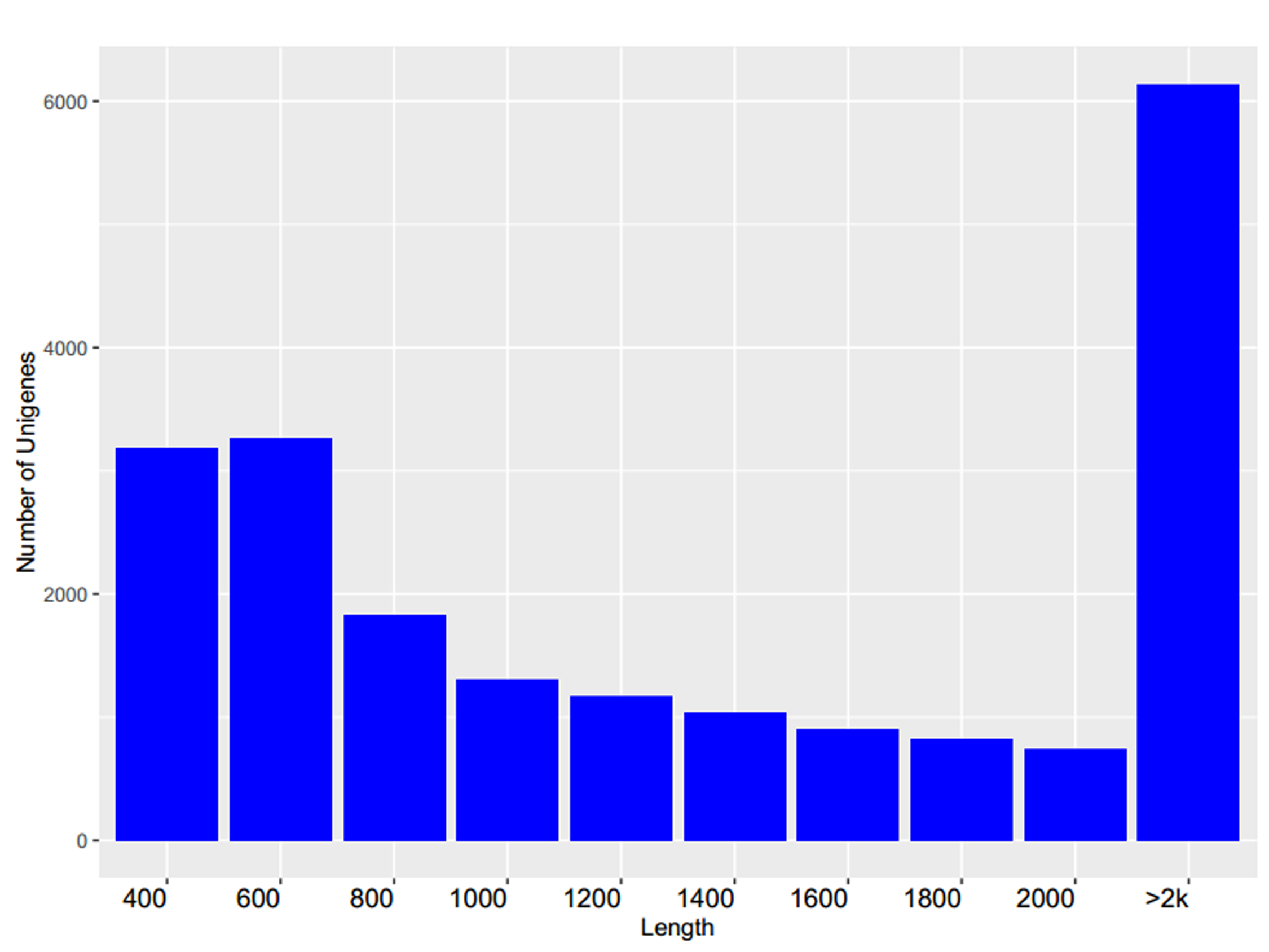

Supplement: S1 Fig — (TIF) [file pone.0179083.s001.tif]

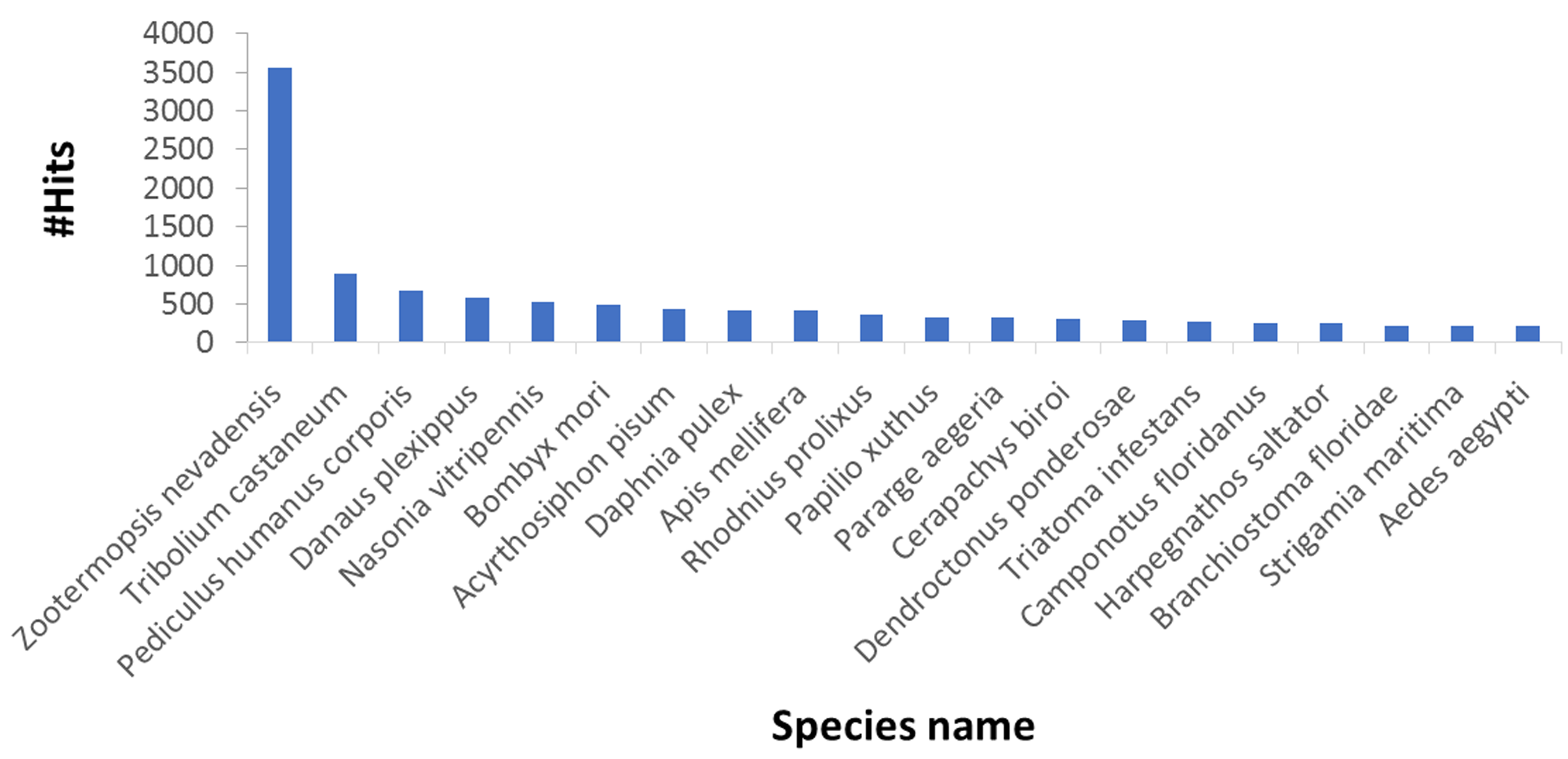

Supplement: S2 Fig — (TIF) [file pone.0179083.s002.tif]

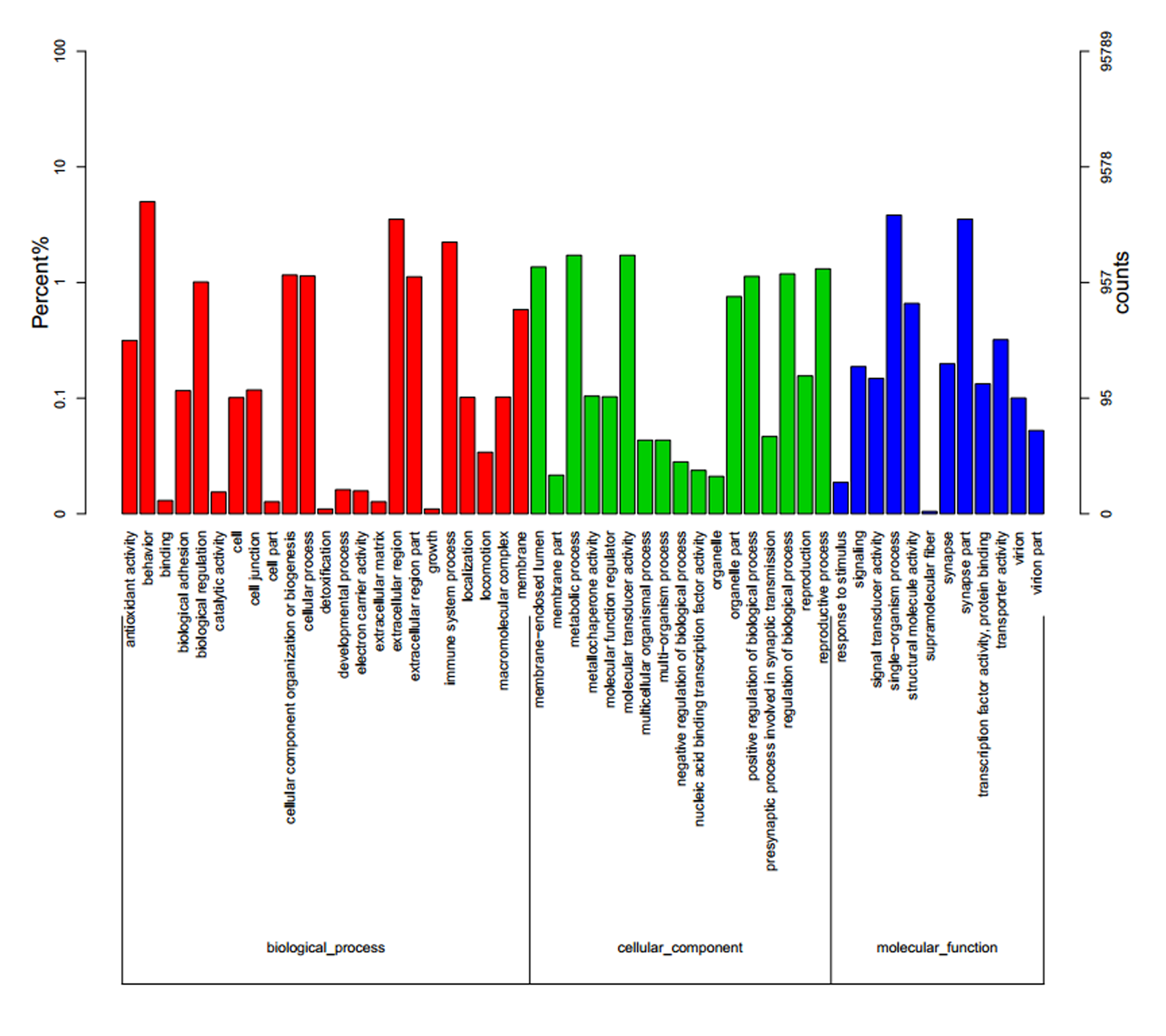

Supplement: S3 Fig — (TIF) [file pone.0179083.s003.tif]

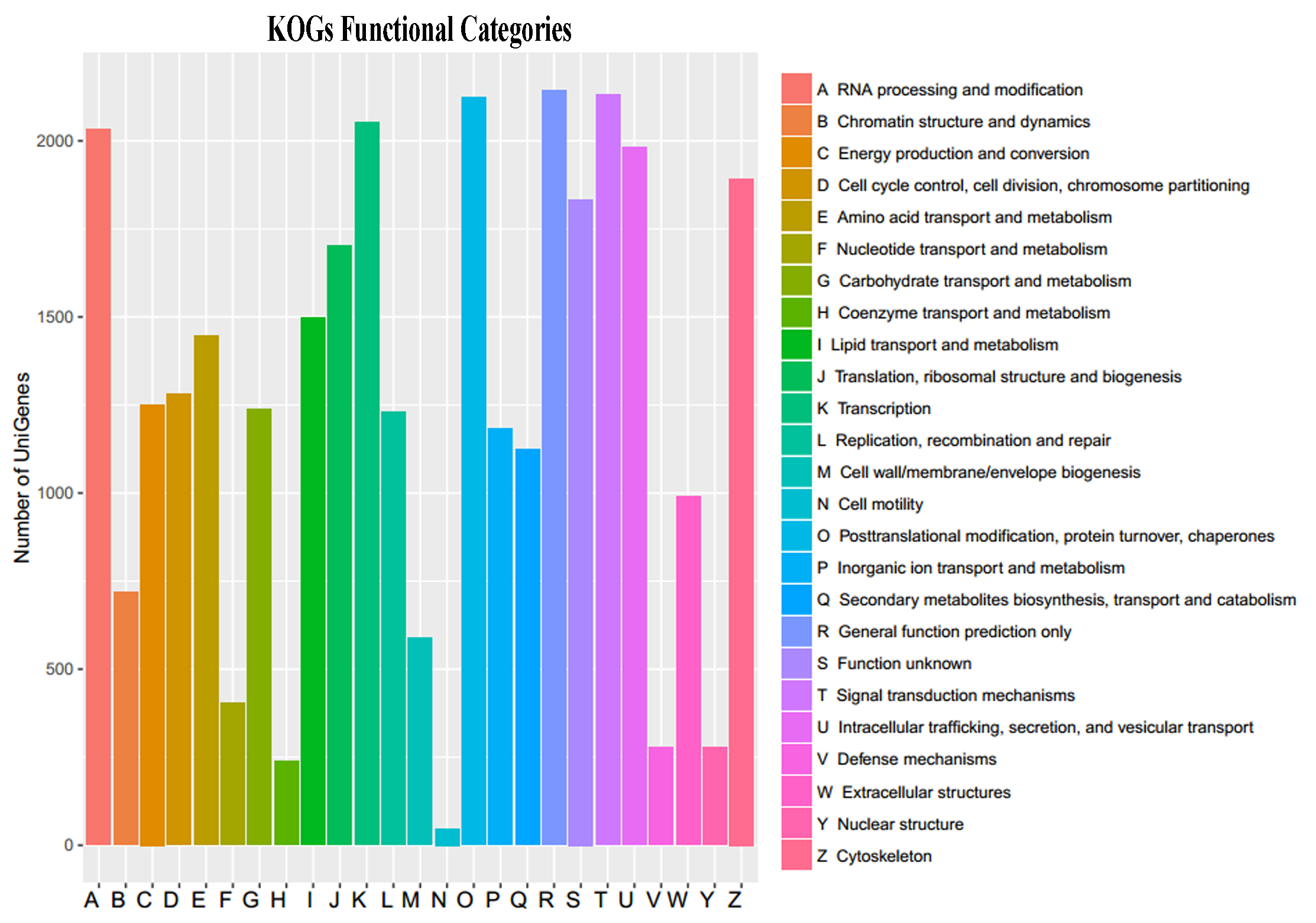

Supplement: S4 Fig — (TIF) [file pone.0179083.s004.tif]

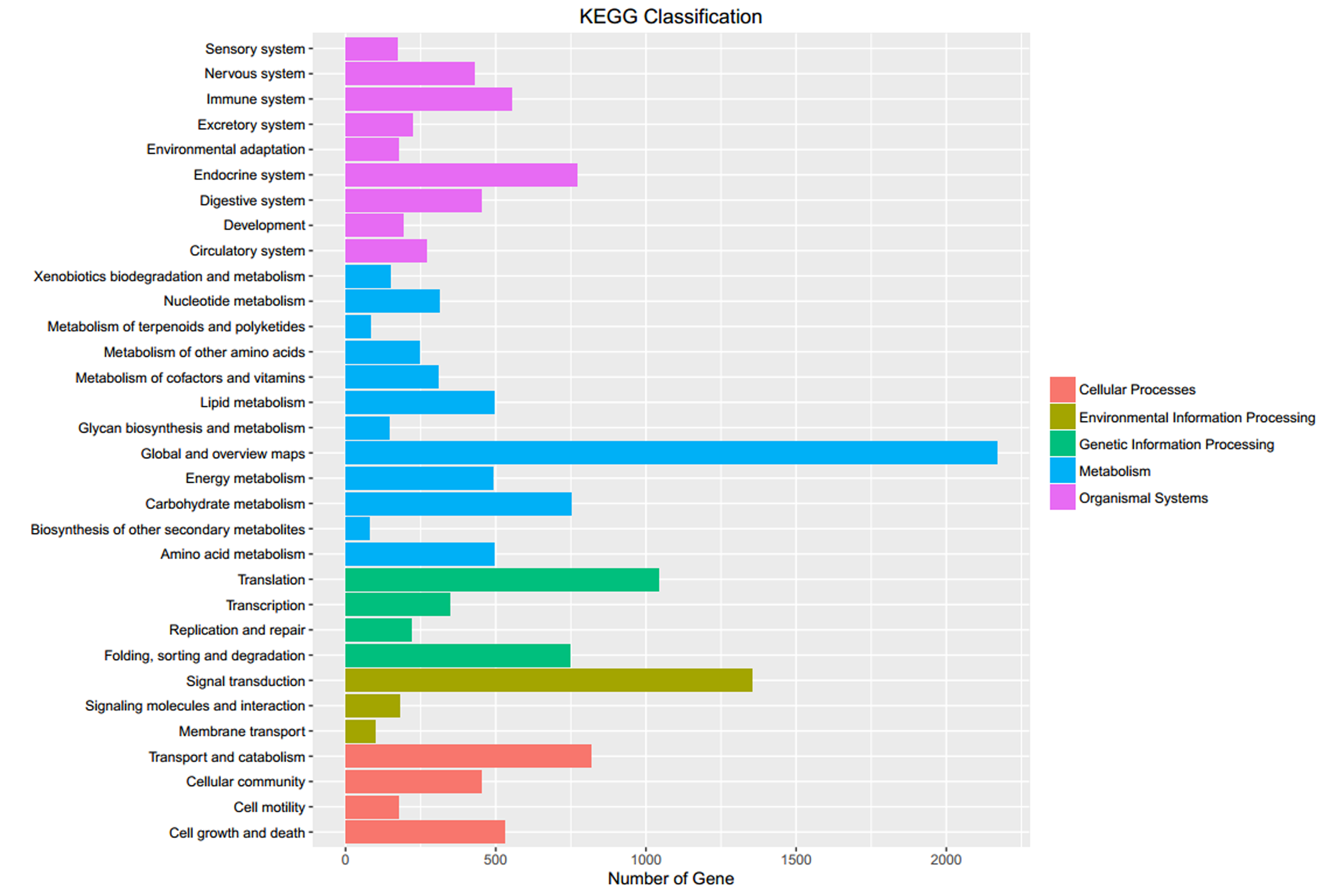

Supplement: S5 Fig — (TIF) [file pone.0179083.s005.tif]

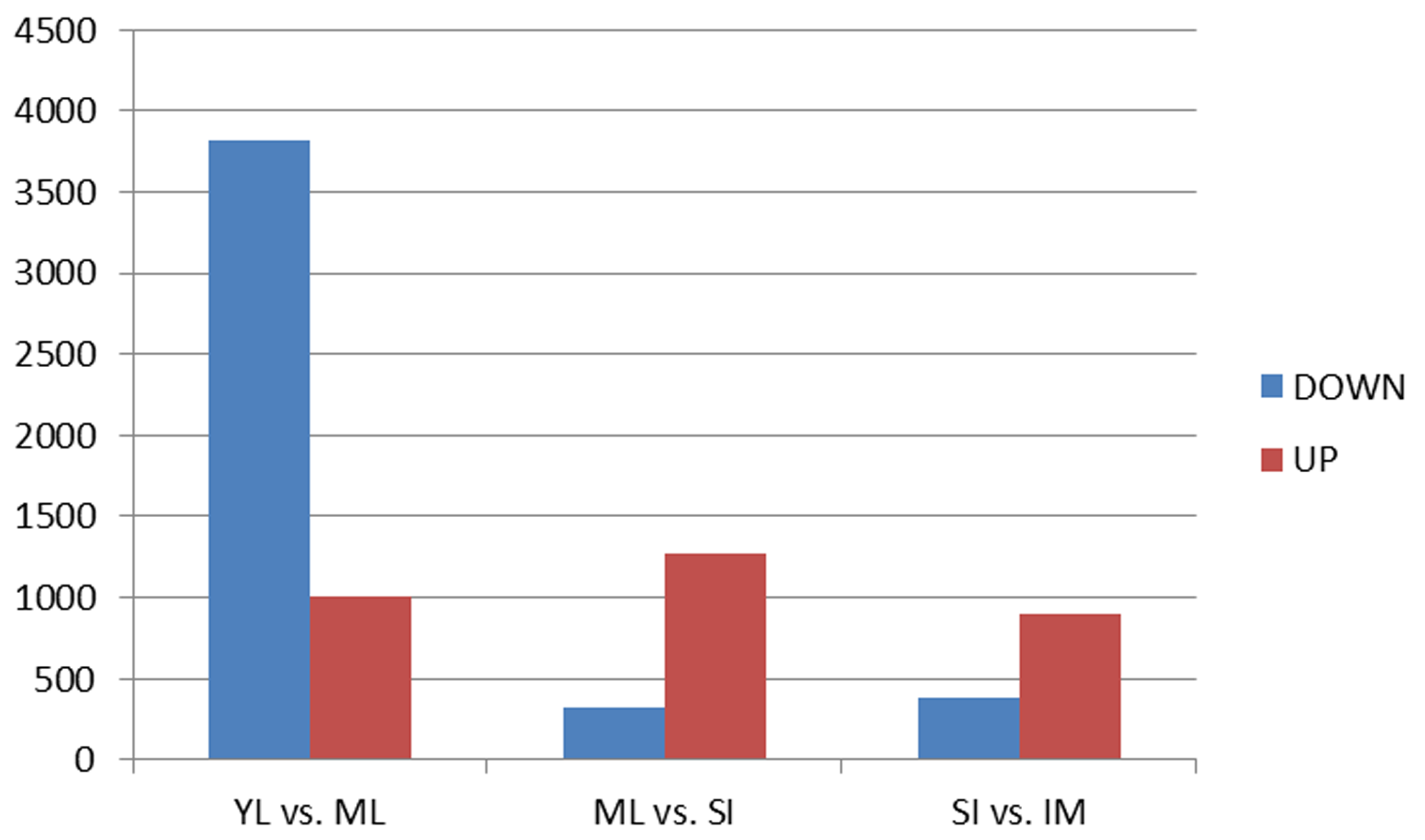

Supplement: S6 Fig — (TIF) [file pone.0179083.s006.tif]
