# Supplementary material for: De novo transcriptome of the mayfly Cloeon viridulum and transcriptional signatures of Prometabola
Source: PLoS One. 2017 Jun 21;12(6):e0179083. doi: 10.1371/journal.pone.0179083 (PMC5479533; doi:10.1371/journal.pone.0179083)
Supplement: S1 Table — (PDF) [file pone.0179083.s007.pdf]

Table S1 Summary of repeats identified in *Cloeon viridulum.*

**Sample Name**	**Seq. type**	**Orientation**	**Raw reads (M)**	**Raw bases(G)**	**Q20 Value (%)**
YL1	mRNA	Forward/Reverse	40.4	5.0	97.2
YL2	mRNA	Forward/Reverse	48.6	6.1	97.5
YL3	mRNA	Forward/Reverse	53.8	6.7	97.4
ML1	mRNA	Forward/Reverse	82.8	10.4	97.4
ML2	mRNA	Forward/Reverse	49.9	6.2	97.7
ML3	mRNA	Forward/Reverse	54.0	6.7	97.8
SI1	mRNA	Forward/Reverse	47.3	5.9	97.7
SI2	mRNA	Forward/Reverse	46.9	5.9	97.5
SI3	mRNA	Forward/Reverse	46.9	5.9	97.5
IM1	mRNA	Forward/Reverse	48.0	6.0	97.5
IM2	mRNA	Forward/Reverse	51.1	6.4	97.5
IM3	mRNA	Forward/Reverse	46.8	5.9	97.4
CG1	mRNA	Forward/Reverse	40.6	5.1	97.6
CG2	mRNA	Forward/Reverse	41.1	5.1	97.6
CG3	mRNA	Forward/Reverse	52.6	6.6	97.5
