# Supplementary material for: De novo transcriptome of the mayfly Cloeon viridulum and transcriptional signatures of Prometabola
Source: PLoS One. 2017 Jun 21;12(6):e0179083. doi: 10.1371/journal.pone.0179083 (PMC5479533; doi:10.1371/journal.pone.0179083)
Supplement: S2 Table — (PDF) [file pone.0179083.s008.pdf]

Table S2 Summary of RNA-seq for YL, ML, SI and IM

Sample ID	Raw reads	Quality trimmed	Adaptor trimmed	Clean reads	Clean ratio
YL1	40,391,604	40,265,308	39,924,673	39,480,392	97.7%
YL2	48,629,644	48,467,935	48,060,623	47,495,824	97.7%
YL3	53,825,052	53,665,929	53,210,038	52,617,800	97.8%
ML1	82,832,970	82,029,032	81,341,286	79,929,850	96.5%
ML2	49,881,106	49,716,104	49,346,780	48,887,932	98.0%
ML3	53,987,044	53,855,965	53,443,562	52,947,784	98.1%
SI1	47,268,616	47,149,314	46,731,846	46,218,348	97.8%
SI2	46,947,608	46,807,744	46,379,684	45,839,790	97.6%
SI3	46,944,166	46,816,385	46,402,644	45,889,952	97.8%
IM1	47,994,992	47,855,070	47,403,392	46,847,562	97.6%
IM2	51,094,508	50,940,456	50,472,990	49,893,992	97.7%
IM3	46,806,440	46,645,795	46,205,408	45,637,216	97.5%
CG1	40,614,636	40,496,928	40,152,369	39,726,222	97.8%
CG2	41,129,230	41,012,530	40,674,416	40,251,914	97.9%
CG3	52,613,262	52,450,453	51,987,587	51,405,156	97.7%

Clean ratio=(Clean reads/Raw reads)%
