# Supplementary material for: De novo transcriptome of the mayfly Cloeon viridulum and transcriptional signatures of Prometabola
Source: PLoS One. 2017 Jun 21;12(6):e0179083. doi: 10.1371/journal.pone.0179083 (PMC5479533; doi:10.1371/journal.pone.0179083)
Supplement: S3 Table — (PDF) [file pone.0179083.s009.pdf]

Table S3 Statistical summary of *C. viridulum* transcriptome for assembling.

Statistics	counts	Total length (bp)	N50 (bp)	Average length	longest (bp)	GC %
Contig	140,513	88,351,518	812	629	15,030	43.8
Scaffold	94,580	80,435,809	1,179	850	22,030	44.0
Final UniGene	81,185	73,440,746	2,992	905	22,030	44.0
