# Supplementary material for: De novo transcriptome of the mayfly Cloeon viridulum and transcriptional signatures of Prometabola
Source: PLoS One. 2017 Jun 21;12(6):e0179083. doi: 10.1371/journal.pone.0179083 (PMC5479533; doi:10.1371/journal.pone.0179083)
Supplement: S4 Table — (PDF) [file pone.0179083.s010.pdf]

Table S4 Blast analysis of non-redundant unigenes against public databases.

Gene_number	UniProt	Nr	GO	KOG	KEGG
81,185	30,027	37,878	11,056	15,763	7,199
100%	36.99%	41.23%	13.62%	19.42%	8.87%
