# Supplementary material for: De novo transcriptome of the mayfly Cloeon viridulum and transcriptional signatures of Prometabola
Source: PLoS One. 2017 Jun 21;12(6):e0179083. doi: 10.1371/journal.pone.0179083 (PMC5479533; doi:10.1371/journal.pone.0179083)
Supplement: S5 Table — (PDF) [file pone.0179083.s011.pdf]

Table S5 Primers used in this study

Name of genes	Forward primer (5′-3′)	Reverse primer (5′-3′)
Glyceraldehyde-3-phosphate dehydrogenase (GAPDH)	AAAACGGCAATCTTCTGTC	GTCAACGACCCCTTCATC
Broad-complex (BR-C)	GAGGGATGACGAGGACTT	ATGGGGTACTCTTGAGCAG
Ecdysone receptor A isoform (EcR-A)	GGGCTGTAAGGGTCGTAG	CATCGTCCACATTCCAGA
Hemolymph juvenile hormone binding protein (JHBP)	CCAGGGGAATAGACGAGT	AGTTGGCAGTGTTGGTGT
Methoprene-tolerant protein (Met)	ATCTTGTTGGTCTCCTTGTC	TTGTCTGCCTTTCGCTAC
Scalloped (Sd)	AGCGTCCATTCTCAAACC	GACCTCAACTCCAACATTACA
Wingless protein (Wg)	TGTTGTGAGGTGGTGAGTG	GATTACGAGTGCGGGAGT
Engrailed (En)	ACGGAATGGTGCTGTGGT	AAGCGGGAGTTTCAGGAGA
Vestigial (Vg)	TCGTCGTTTCCAGGTTCAG	TCCAGCCCCATTTCGTTA
Broad-complex core protein isoform 6 (BR-C Z6)	GTCAGTCTTTCTTCCTCCAC	AATCCTTGTCAGCACCCT
Broad-complex, isoform Z1 (BR-C Z1)	GAGAGGGCTTGGTTATCG	GCTGTTGAGGGTGCTGTA
Chitin synthase (CS)	TCGGGTGCGTCCTTTGTA	TCCTCCCCTTGATCGTATTG
Chitinase (CA)	GCCCGTTATTCGTCGTCTC	ACCTGGATTGGATCGCTGT
Ecdysone 20-monooxygenase (20E)	TCGTAAAGTTCCTCCTCAGCC	CCCACCCCAATGTTCAGC
Ecdysone-inducible protein E75 (E75)	GGGGACGGCATTTGGATT	CTCGAGGACGAACGC
Kruppel-like protein 1 (Kr-h1)	GCCACAAGTCCTTTTCCGTC	TGTGCAGCTTCCCTGAGTGT
Myosin heavy chain (MHC)	GCTTTTGGGCTGCGACTG	GGTGACCTGATCCTTGTTACGG
Chitinase-3-like protein (CHI3L)	GAGGGCGGTGCTCTGCTT	CATCTGCGGAGGTGGAGTC
Nuclear hormone receptor HR3 (HR3)	ATCTCCGCCACTCCTTTCA	GAGGATGAGCCGTTACTTCG
TGF-beta receptor type-1 (TGF-βR T1)	TGAGACACGACAACATCCT	TAGTCAAACAGCGAGCCA
Hedgehog protein (Hh)	GACTTGACGGAGCAGTGGAT	ACATCACGACATCGGACAGAG
